# Stability of CoP_*x*_ Electrocatalysts in Continuous and Interrupted Acidic Electrolysis of Water

**DOI:** 10.1002/celc.201701119

**Published:** 2018-02-22

**Authors:** Andrey Goryachev, Lu Gao, Yue Zhang, Roderigh Y. Rohling, René H. J. Vervuurt, Ageeth A. Bol, Jan P. Hofmann, Emiel J. M. Hensen

**Affiliations:** ^1^ Laboratory of Inorganic Materials Chemistry, Department of Chemical Engineering and Chemistry Eindhoven University of Technology P.O. Box 513 5600MB Eindhoven The Netherlands; ^2^ Laboratory of Plasma and Materials Processing, Department of Applied Physics Eindhoven University of Technology P.O. Box 513 5600MB Eindhoven The Netherlands

**Keywords:** cobalt phosphide, stability, hydrogen evolution reaction, OLEMS/DEMS, model electrodes

## Abstract

Cobalt phosphides are an emerging earth‐abundant alternative to platinum‐group‐metal‐based electrocatalysts for the hydrogen evolution reaction (HER). Yet, their stability is inferior to platinum and compromises the large‐scale applicability of CoP_*x*_ in water electrolyzers. In the present study, we employed flat, thin CoP_*x*_ electrodes prepared through the thermal phosphidation (PH_3_) of Co_3_O_4_ films made by plasma‐enhanced atomic layer deposition to evaluate their stability in acidic water electrolysis by using a multi‐technique approach. The films were found to be composed of two phases: CoP in the bulk and a P‐rich surface CoP_*x*_ (P/Co>1). Their performance was evaluated in the HER and the exchange current density was determined to be *j*_0_=−8.9 ⋅ 10^−5^ A/cm^2^. The apparent activation energy of HER on CoP_*x*_ (*E*_a_=81±15 kJ/mol) was determined for the first time. Dissolution of the material in 0.5 M H_2_SO_4_ was observed, regardless of the constantly applied cathodic potential, pointing towards a chemical instead of an electrochemical origin of the observed cathodic instability. The current density and HER faradaic efficiency (FE) were found to be stable during chronoamperometric treatment, as the chemical composition of the HER‐active phase remained unchanged. On the contrary, a dynamic potential change performed in a repeated way facilitated dissolution of the film, yielding its complete degradation within 5 h. There, the FE was also found to be changing. An oxidative route of CoP_*x*_ dissolution has also been proposed.

## 1 Introduction

Hydrogen is one of the most important inorganic base chemicals used in industrial processes, such as ammonia synthesis and petroleum refining. It is also a promising clean energy carrier to replace fossil‐derived fuels in the near future.^1^ Currently, the dominant technology for hydrogen production is steam reforming of hydrocarbons, which is neither environmentally friendly nor sustainable.^2^ Hydrogen can also be produced by electrochemical water splitting, wherein water is decomposed to hydrogen and oxygen by electrolysis.^3^ Combined with renewable electricity produced from solar or wind power, electrochemical water splitting could be a clean and sustainable approach to producing hydrogen.^4^,^5^

Electrocatalysts play a critical role in the performance of electrochemical water splitting devices.^6^,^7^ High energy efficiency for water splitting can only be achieved if suitable electrocatalysts are employed to drive the hydrogen evolution (HER) and oxygen evolution reactions (OER). The main criteria of a good HER catalyst are the ability to moderately bind both H^+^ and atomic H, while still being able to efficiently desorb H_2_.^8^ Platinum is the best‐studied and best‐performing catalyst for HER and requires only a very small overpotential, even at high current densities in acidic solutions. However, the scarcity and high cost of Pt limits its large‐scale applicability. The search for active and stable earth‐abundant electrocatalysts to replace precious metals has triggered a lot of research efforts. The majority of transition metals (e. g. Cr, Mo, Fe, Co, Ni) bind protons too strongly or too weakly and thus are inactive in HER if present solely. However, addition of phosphorus leads to an increase in performance of those metals by stabilization of the metal d‐orbitals and a small metal to P charge transfer (i. e. ligand effect).^9^ For first‐row transition metal phosphides (TMP) it was shown that P (H^+^ acceptor) “dilutes” metal atoms (hydride acceptors) resulting in a weakening of the M−H bond (i. e. ensemble effect).^8^,^10^,^11^ Moreover, it was also found that an increasing P/metal ratio of TMP yields an increase in HER performance which is also reflected in decreasing Tafel slopes.^10^,^12^,^13^ Thus, transition metal phosphides, including nickel phosphides,^14^, ^15^, ^16^ cobalt phosphides,^17^, ^18^, ^19^, ^20^, ^21^, ^22^ iron phosphides,^23^, ^24^, ^25^ molybdenum phosphides,^10^,^26^,^27^ tungsten phosphides,^28^,^29^ copper phosphides^30^ and their alloys^31^, ^32^, ^33^ have emerged as potential HER electrocatalysts. In order to increase the apparent electrochemical activity, porous conductive substrates such as carbon cloths and nickel foams are often used as current collectors. Since it is difficult to exactly determine the real electrochemical surface area, most of the reported current densities are normalized by the geometric surface area of electrodes. This approach, however, does not include the variation in catalyst loading or electrochemical surface area.^31^ For this reason, it is preferable to perform electrocatalytic performance evaluation on flat substrates to obtain accurate values of the intrinsic activities.^34^ In terms of stability testing, high catalyst loadings may result in artificially prolonged lifetimes. Therefore, a low mass loading is preferably used when testing the stability of electrocatalysts.^34^ Stability tests are usually done by either chronoamperometry/chronopotentiometry (CA/CP) measurements or repeated linear scan/cyclic voltammetry (LSV/CV) experiments. CA/CP shows the stability under constant and continuous working conditions, while repeated LSV/CV demonstrates the stability under dynamic power supply. The latter is especially important when a water splitting device is coupled with renewable (e. g. solar or wind) electricity, which is intermittent in nature.^35^ Besides electrochemical methods, compositional analysis of both electrodes (by X‐ray Photoelectron Spectroscopy, XPS) and the electrolytes (Inductively coupled plasma mass spectrometry, ICP‐MS) after the stability tests is also essential to evaluate the stability of electrocatalysts.^36^

In the present work, we studied the intrinsic HER activity and stability of CoP_*x*_ in acid electrolyte. We deposited 20 nm thick cobalt oxide films on flat Au‐coated Si substrates by plasma enhanced atomic layer deposition (PE‐ALD). We chose PE‐ALD to deposit cobalt oxide films because of its low processing temperatures, sub‐nm thickness control and 3D conformity. Then, we converted the cobalt oxide films to cobalt phosphide (CoP_*x*_) by gas‐phase phosphidation. With such a low mass loading on a flat surface, we were able to study the intrinsic stability and activity of CoP_*x*_. The apparent stability of CoP_*x*_ measured by repeated LSV is much lower than that measured by CA. Combining these results with compositional analysis of both electrodes and electrolytes after stability testing, we propose a possible degradation mechanism of cobalt phosphide electrodes in acidic electrolytes. We also studied the temperature‐dependent kinetics from which the apparent activation energy for HER over CoP_*x*_ in acidic electrolyte has been derived. In particular, On‐Line Electrochemical Mass Spectroscopy (OLEMS) was employed to follow gaseous products evolution simultaneously to the applied electrochemical treatment. Combined EC‐OLEMS measurements are commonly used in the field of electrochemistry for an accurate evaluation of electrode performance, stability, selectivity and reaction kinetics.^37^, ^38^, ^39^, ^40^, ^41^, ^42^ From our studies, we emphasize the importance of using flat thin film model electrocatalyst and compositional analysis of the electrode, electrolyte as well as gaseous products to characterize the intrinsic stability and activity of earth‐abundant electrocatalysts.

## 2 Results and Discussion

### 2.1 CoP_*x*_ Preparation and Characterization

CoP_*x*_ films were thermally prepared by phosphidation of smooth PE‐ALD fabricated Co_3_O_4_ films with an initial thickness of 20 nm. The Co 2p XP spectrum of as‐prepared cobalt oxide (Figure S1a) confirms its chemical composition by showing the characteristic Co_3_O_4_ profile and the matching binding energy (BE) positions of spectral lines (BE(Co 2p_3/2_)=779.6 eV) and shake‐up satellites (BE(Co 2p_3/2,sat._)=789.9 eV).^43^ Moreover, the survey XP spectrum shows the absence of substrate‐related peaks (e. g. Au/Ti/Si) and contaminations other than adventitious carbon (Figure S1b). Thus, we conclude that PE‐ALD yields conformal Co_3_O_4_ films with a uniform coverage. Additionally, atomic force microscopy (AFM) shows that the average surface roughness of the as‐deposited Co_3_O_4_ film is *σ*=0.7 nm – the same as for the bare Au/Ti/Si substrate (Figure S1c, d).

**Figure 1:**
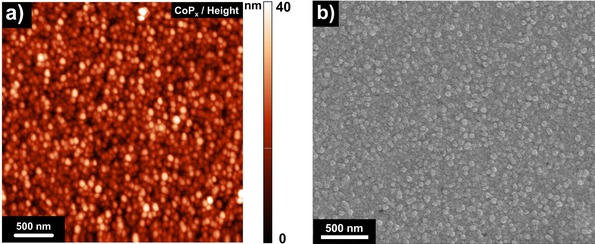
a) Atomic force and b) scanning electron micrographs of fresh CoP_*x*_.

Upon thermal phosphidation treatment conducted at temperatures ranging from 350–450 °C, Co_3_O_4_ undergoes both chemical and morphological changes. The morphology analysis of the CoP_*x*_ film (400 °C) shows that the film is composed of spherical particles with an average lateral size of 84±10 nm and an average height of 18±4 nm (Figure 1a, b). We have to take into account that the real shape of thermally prepared CoP_*x*_ particles may differ from what is observed by AFM, since the in‐plane accuracy of AFM is influenced by the tip curvature. The average surface roughness of the phosphidized film increases to 4.5 nm which is 6 times higher than the roughness of the initial Co_3_O_4_ film. This fact together with the appearance of Au 4f peaks in the XP spectrum of CoP_*x*_ (Figure S2a) shows that phosphidation of Co_3_O_4_ involves agglomeration of the particles composing the initial film.

The XRD pattern of the untreated oxide film reveals the presence of two weak reflections attributed to Co_3_O_4_ (111) and (511), while other reflections are either not observable or overlapping with substrate peaks (Figure S2b). Phosphidation leads to disappearance of oxide reflections, although no visible reflections attributed to any other new phase (e. g. CoP, Co_2_P, etc.) were found to be formed. This can be explained by both low thickness and crystallinity of the phosphide film formed during the relatively mild thermal phosphidation. Thus, additional characterization was performed to determine the structural and chemical composition of the CoP_*x*_ film.

**Figure 2:**
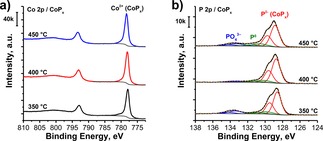
a) Co 2p and b) P 2p XP spectra of CoP_*x*_ electrodes prepared at different phosphidation temperatures.

XPS analysis of the Co 2p spectral region reveals the formation of a dominant P‐rich CoP_*x*_ phase (BE(Co 2p_3/2_)=778.2±0.2 eV) in which Co is assumed to be mostly covalently bound to P with corresponding charges δ^+^ (∼1+) and δ^−^ (∼1−), respectively (Figure 2a).^44^, ^45^, ^46^ The small shoulder at higher binding energies (BE(Co 2p_3/2_) >779.6 eV) is most likely attributed to a native oxide formed on the surface of CoP_*x*_ upon air exposure. The P 2p XP spectral region reveals that the major (76±4 %) fraction of the surface phosphorous is present in P^δ−^ state (BE(P 2p_3/2_)=128.6±0.1 eV) with some part of phosphide being oxidized to form a mixture of P^0^ (BE(P 2p_3/2_)=130.2±0.2 eV) and PO_4_^3−^ (BE(P 2p_3/2_)=133.3±0.1 eV) (Figure 2b). Furthermore, P to Co ratios were derived from the corresponding spectral regions and found to be similar regardless of the phosphidation temperature (Table 1). The electrochemical performance of the CoP_*x*_ electrodes was also found not to be influenced by phosphidation temperature (Figure S3a). Thus, all further measurements were conducted on the sample prepared at 400 °C.

**Table 1:** P to Co ratio derived from Co 2p and P 2p XPS analysis.

P/Co ratios from XPS	Overall P content	P^δ−^ content
*T*=350 °C	4.1	3.0
*T*=400 °C	3.9	3.2
*T*=450 °C	4.0	2.9
average ratio	4.0±0.1	3.0±0.1

**Figure 3:**
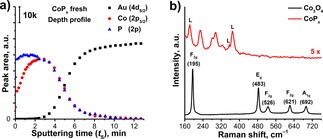
a) XPS depth profile of fresh CoP_*x*_; b) Raman spectra (*λ_ex_*=632.8 nm) of as‐prepared Co_3_O_4_ (black) and thermal CoP_*x*_ (red) films, L marks impurity lines of the HeNe laser.

The bulk structure of CoP_*x*_ has been further studied by XPS depth profiling (DP‐XPS). Depth profiling of the fresh sample points to a difference between surface and bulk composition of CoP_*x*_ (Figure 3a). Particularly, a gradual decrease in the P to Co ratio was observed upon sputtering, arriving at a constant 1 : 1 composition (CoP) at *t*_S_>2.4 min (i. e. in the bulk). Although, cobalt phosphides normally crystalize in the form of CoP and Co_2_P – the formation of P‐rich polyphosphides is a known phenomenon in phosphorous chemistry.^47^,^48^ This can be reasoned by a diffusion limitation during the phosphidation reaction resulting in an increasing contact time between PH_3_ and lower phosphides in the top layer of the film. Thus, we assume that formation of polyphosphides as well as other P‐containing species (e. g. P^0^) is the reason for the observed phosphorus excess. Unfortunately, an exact thickness analysis of DP‐XPS requires information on the sputtering rates of the studied materials, which is unknown for CoP_*x*_. Thus, we could only estimate the percentage of the P‐rich fraction (*t*_S_=2.4 min, 22.8 %) in the overall CoP_*x*_ film (*t*_S_=10.5 min, 100 %). Qualitative analysis of DP‐XPS reveals the presence of oxidized Co species in the bulk (*t*_S_=4 min) which can be attributed to unreacted Co_3_O_4_ (Figure S4a, b). Together with that, the presence of P^0^ and residuals of CoPO_*x*_ were also observed.

The Raman spectrum of the as‐prepared PE‐ALD film also confirms its Co_3_O_4_ structure (Figure 3b).^49^ Phosphidation leads to the disappearance of the oxide‐related peaks (195, 483, 526, 621 and 692 cm^−1^) and the appearance of a new set of peaks corresponding to CoP_*x*_ (230, 280, 295 and 320 cm^−1^) and HeNe laser impurities (180, 355 and 370 cm^−1^, *cf*. Figure S2c). The Raman peaks, which we attributed to CoP_*x*_, match with the Raman spectrum of electrodeposited CoP/CoP_*x*_O_*y*_ mixed electrodes reported by Saadi et al.^22^ There, the question of the exact composition of the Raman‐active phase remained unanswered. As there is no Raman data on pure CoP available in literature, we synthesized bulk CoP by thermal phosphidation of Co_3_O_4_ powder (*cf*. XRD pattern (Figure S5a) allowing us to measure the Raman spectrum of CoP (Figure S5b). Based on this, we assign CoP to be the major component in the bulk of the CoP_*x*_ film.

Additionally, we utilized DFT calculations to obtain a simulated Raman spectrum of CoP. Calculations were done on a Co_8_P_8_ cluster with the structure depicted in Figure S6a matching literature data.^50^ The computed spectrum does show similar features as compared to the measured spectrum (Figure S6b), particularly the peaks at 250, 295, 310, and 330 cm^−1^. We notice a consistent but small shift of about 10–20 cm^−1^ to higher wavenumbers for every peak in the computed spectrum as compared to the measured spectrum. This can be explained by methodological differences between VASP and Gaussian software utilized for structural optimization and Raman spectrum simulation, respectively. Moreover, the computations were performed on an isolated cluster leading to different electronic properties as compared to the actually measured bulk CoP phase. However, we aim at a qualitative description of the Raman spectrum and the correspondence between the measured and computed spectrum is considered to be good.

### 2.2 Performance of CoP_*x*_ as a HER Electrocatalyst

In order to evaluate the HER performance of the as‐synthesized CoP_*x*_ films, faradaic efficiency (FE), the exchange current density and the apparent activation energy were measured by CA and OLEMS. For this purpose, a series of potential pulses with a duration of 10 s was applied and both current density and *m/z*=2 (H_2_^+^) ion current were recorded simultaneously. Pulsed chronoamperometric measurements were chosen over continuous voltammograms since it enables longer accumulation of reaction products which is especially crucial for OLEMS measurements of FE at lower overpotentials.

The amounts of evolved H_2_ which are proportional to *m/z*=2 peak area were plotted versus values of negative charge generated during the pulses to make sure that faradaic efficiency is not changing during the experiment (Figure S7a). In the case of the *T*‐dependent pulse CA treatment of CoP_*x*_ cathodes accompanied by in‐between protective potentials (*E*=−0.12 V, 40 min), a single Faraday slope was observed. Thus, the FE of HER on CoP_*x*_ was found to be constant within ±10 % under the chosen experimental conditions, while in a reference experiment, where no protective currents were applied between the temperature steps, a clear alteration of the Faraday slopes could be observed (Figure S7b).

**Figure 4:**
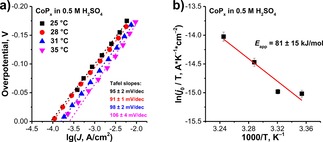
*T*‐dependent Tafel plots and b) corresponding Arrhenius plot of HER conducted on CoP_*x*_ in 0.5 M H_2_SO_4_ with intermittent cathodic protection.

The obtained *T*‐dependent *I–V* curves were converted to Tafel plots in order to assess the kinetics of the HER on CoP_*x*_ (Figure 4a). The Tafel slope was found to be slightly increasing with temperature, reaching a value of 95±2 mV/dec and 106±4 mV/dec at 25 °C and 35 °C, respectively. Based on the Tafel slope one can estimate the reaction mechanism, which, in case of HER, can be a combination of either Volmer/Tafel or Volmer/Heyrovsky reactions: Equations (1) and (3) or (1) and (2), respectively.^51^ However, an analysis of the Tafel slope values alone should be treated with care, since it cannot provide a conclusive proof of a certain reaction mechanism without additional experiments, although it can indicate possible rate‐limiting processes. In our case, the large value of the Tafel slope suggests a Volmer‐type reaction (i. e. proton discharge) to be the rate‐determining step. This can be attributed to the limited number of proton adsorption sites, which was also reported to be the case for MoS_2_ cathodes.^52^,^53^ Indeed, lower Tafel slope values *(∼*60 mV/dec) can be achieved on nanostructured CoP_*x*_ cathodes with high surface area, while flat electrodes had similar values to what is reported in our study.^34^

(1) $$H{}_{3}O{}^{+}+ϒ{}_{\mathrm{ad}}+e{}^{-}\to H{}_{\mathrm{ad}}+H{}_{2}O\;\;\;\;\left(120\;\mathrm{mV}/\mathrm{dec}\right)$$

(2) $$H{}_{\mathrm{ad}}+H{}_{3}O{}^{+}+e{}^{-}\to ϒ{}_{\mathrm{ad}}+H{}_{2}↑+H{}_{2}O\;\;\;\;\left(40\;\mathrm{mV}/\mathrm{dec}\right)$$

(3) $$H{}_{\mathrm{ad}}+H{}_{\mathrm{ad}}\to ϒ{}_{\mathrm{ad}}+ϒ{}_{\mathrm{ad}}+H{}_{2}↑\;\;\;\;\left(30\;\mathrm{mV}/\mathrm{dec}\right)$$

In addition to the Tafel slope values, the exchange current densities (*j*_0_) of the *T*‐dependent measurements were determined by extrapolation of the linear Tafel region to *η*=0 V. *j*_0_ at 25 °C (8.9 ⋅ 10^−5^ A/cm^2^) was found to be comparable with reported values for CoP (0.4–1.2 ⋅ 10^−4^ A/cm^2^).^54^, ^55^, ^56^ Changing temperature resulted in an expected increase of the exchange current densities (i. e. 9.4 ⋅ 10^−5^ (28 °C), 1.6 ⋅ 10^−4^ (31 °C) and 2.5 ⋅ 10^−4^ (35 °C) A/cm^2^). The *j*_0_ values were plotted in the form of an Arrhenius plot (Figure 4b) to determine the apparent activation energy (*E*_app_) of the HER, based on Equation (4), where *k_B_* and *h* are the Boltzmann and Planck constants, *z* and *e* are the number of electrons and the elementary charge and *N*_as_ is the number of surface active sites.

(4) $${ln(j}_{0}/T)=\alpha -\frac{E_{app}}{k_{B}T},\;\;\;\alpha =\frac{k_{B}zeN_{as}}{h}$$

The *E*_app_ of HER on CoP_*x*_ was found to be 81±15 kJ/mol. To the best of our knowledge, the *E*_app_ value of TMP cathodes is reported here for the first time and can potentially be used in follow‐up theoretical studies on the system.

Although, the HER performance of CoP_*x*_ does not surpass Pt (*j*_0_≈10^−3^ A/cm^2^, *E*_app_≈30 kJ/mol),^57^,^58^ it is still one of the best‐performing non‐noble metal based catalysts for electrocatalytic HER. In order to increase the performance of the CoP_*x*_ electrocatalyst, different strategies could be applied, including an increase of the P content in the material (e. g. CoP_3_), an introduction of unsaturated P sites and nanostructuring of the surface.

### 2.3 Stability of CoP_*x*_ under Acidic HER Conditions: Continuous vs. Intermittent Operation

In order to assess the stability of CoP_*x*_ films under HER conditions, a series of experiments with different types of electrochemical treatments was performed. At first, the stability of CoP_*x*_ under continuous operation was evaluated by subjecting the electrode to a constant potential (*E*=−0.12 V) for 5 h (CA, chronoamperometry).

This potential value was chosen since it was found to be negative enough to assure a decent rate of H_2_ production (*η*_HER_=−90 mV, Figure S3b) but did not lead to an overproduction of H_2_ bubbles which would disturb the measured current. In the second experiment, a series of repeated linear cathodic sweeps (+0.24 V→−0.26 V, 20 mV/s) was applied to a fresh CoP_*x*_ sample (rLSV). The starting potential was set equal to the open circuit potential (OCP) of fresh CoP_*x*_ in order to avoid anodic currents, while the end potential lies within the Tafel region of the voltammogram. rLSV treatment allows evaluation of the electrode stability under fast potential scanning (20 mV/s).

**Figure 5:**
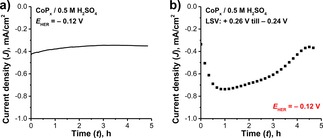
a) Chronoamperograms of HER at constant potential (*E*_HER_=−0.12 V); b) *J‐t* profile of rLSV‐treated CoP_*x*_ cathode where *J* values were sampled from the corresponding voltammograms at *E*_HER_=−0.12 V.

Analysis of the chronoamperogram obtained in the CA experiment reveals an apparent stability of CoP_*x*_ under constant cathodic polarization. Only a slight current decrease (17 %) was observed in the first 2 hours of electrolysis arriving at a plateau current density of −0.35 mA/cm^2^ (Figure 5a).

On the other hand, the currents derived from the rLSV experiment (Figure 5b) point to dynamic transformations of the electrode surface initiated by the applied treatment, even though the voltammograms of CoP_*x*_ do not contain any features related to surface reduction or anodic currents (Figure S3a). In about one hour, the cathodic current increased and reached its maximum value of −0.74 mA/cm^2^. After passing this point, the trend changed to the opposite and the cathodic current began to decrease, eventually arriving at a constant value of −0.37 mA/cm^2^ (50 % less than maximum value) at 4.3 h. Together with that, unlike in the CA case, the appearance of the Au layer (i. e. substrate) was observed visually and confirmed by XPS after 5 h of rLSV treatment (Figure S8a). In the case of CoP_*x*_ electrodes, a cathodic current increase can potentially be attributed to a change of HER faradaic efficiency, electrode dissolution or capacitive charging of the surface. Structural and compositional changes of the spent electrodes caused by the performed electrochemical treatments were analyzed by a comprehensive set of characterization techniques.

**Figure 6:**
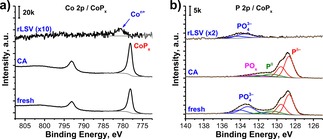
a) Co 2p and b) P 2p XP spectra of CoP_*x*_ electrodes subjected to rLSV and CA treatments.

Analysis of the Co 2p XP spectra of the CA‐treated CoP_*x*_ electrode reveals only a slight change in intensity of the Co^δ+^ peak (BE(Co 2p_3/2_)=778.1 eV), while the position and line shape were found to be preserved (Figure 6a). Thus, one can assume that the chemical state of cobalt is preserved during continuous HER under acidic conditions. However, if the sample was subjected to rLSV treatment, almost complete disappearance of the Co^δ+^ peak was observed. Although, some minor amount of cobalt was still present on the surface in form of oxidized cationic species (CoPO_4_ or CoO_*x*_) appearing at higher BE (780.6 eV). A similar trend was found in the P 2p spectral region (Figure 6b).

The sample subjected to the CA treatment shows a similar amount of overall P species compared to the fresh sample, with a slight change in the composition of the native oxide. Particularly, the P 2p spectrum of the CA‐treated CoP_*x*_ has a new PO_*x*_ component with a BE(P 2p_3/2_)=132.5 eV (oxidation state of P is below 5+) while the fresh sample had mainly PO_4_^3−^ species with a higher BE value (133.3 eV). Other components of P 2p spectra are matching with the fresh sample (P^δ−^: 128.7 eV and P^0^: 130.8 eV). In contrast, the rLSV‐treated sample shows the absence of phosphide‐related spectral components with only a minor amount of residual PO_4_^3−^ (133.6 eV), supporting the statement that most of the CoP_*x*_ film was dissolved during rLSV treatment.

In order to estimate CoP_*x*_ dissolution rates, ICP‐OES measurements of spent electrolytes were performed. These measurements reveal the presence of Co ions in all tested solutions, meaning that electrodes were corroded to a certain degree regardless the type of applied EC treatment. However, the P content could not be evaluated accurately due to the much lower detection limit of phosphorus in ICP‐OES. In the case of the rLSV‐treated electrode, the content of Co^n+^ ions was found to be 181±6 nmol/cm^2^ which can roughly be attributed to the overall Co content of the initial CoP_*x*_ film. This was also confirmed by XPS depth profiling (Figure S8b) which only shows negligible amounts of Co and P present on the surface of the rLSV‐treated electrode.

**Figure 7:**
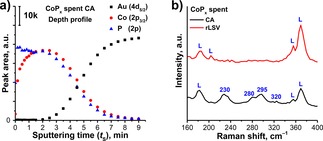
a) XPS depth profile of CA‐treated CoP_*x*_ and b) Raman spectra of CoP_*x*_ cathodes subjected to rLSV (red) and CA (black) treatments, where L marks peaks of the laser impurities.

During the CA treatment, the amount of dissolved Co ions decreased significantly (13±1 nmol/cm^2^), which is only 7.2±0.8 % of the cobalt released in the rLSV experiment. XPS depth profiling of this sample also shows a slight decrease in the amount of the P‐rich fraction (sputter time, *t*_S_=1.3 min) compared to the fresh sample (*t*_S_=2.4 min) (Figure 7a). Moreover, the thickness of the CA‐treated electrode was found to be approximately 14 % less than that of the fresh electrode (9 min vs. 10.5 min *t_S_* to fully remove CoP_*x*_ layer). The Raman spectrum of the CA‐treated electrode suggests preservation of the CoP_*x*_ structure with only a change in intensity of the peak at 320 cm^−1^ (Figure 7b, black). However, the Raman spectrum of the rLSV‐treated sample shows the absence of CoP_*x*_ and Co_3_O_4_ related peaks (Figure 7b, red), in line with the ICP‐OES and XPS measurements.

To assess the temporal evolution of Co leaching during CA treatment at *E*=−0.12 V, the electrolyte was sampled after 2.5, 5.0, 7.5 hours and analyzed by *ex‐situ* ICP‐OES (Figure S9). The dissolution rate at the initial stage of electrolysis (*t*<2.5 h) was not accessible in this way due to the low concentration of Co ions in the electrolyte. We found that Co leaching occurred throughout the entire experiment with a slightly higher rate (∼17 %) in the first sampled period (2.5 hours) of electrolysis. High initial dissolution rates typically originate from dissolution of native oxides and can be quantified by e. g. online ICP‐OES or ‐MS analysis.^36^,^59^ Unlike for CoP, the initial dissolution is shown to contribute the most to the measured dissolution rates of Co_2_P, while following potentiostatic treatment led to almost zero Co leaching.^36^

Additionally, in order to evaluate the stability of CoP_*x*_ under resting conditions (i. e. chemical stability), a reference experiment was conducted in which a fresh CoP_*x*_ substrate was immersed in 0.5 M H_2_SO_4_ solution for 5 h without any additional EC treatment. A constant dissolution rate of CoP_*x*_ (Co, 2.4±0.1 nmol ⋅ h^−1^ ⋅ cm^−2^) was found which is comparable with that for the CA‐treated sample (Co, 2.6±0.2 nmol ⋅ h^−1^ ⋅ cm^−2^). From this, we can conclude that CoP_*x*_ dissolution, observed in the case of the CA treatment, proceeds mostly via a chemical route with additional involvement of anodic oxidation during the HER in accordance with the mixed potential theory.^60^ However, it is also obvious that an rLSV treatment of the electrodes leads to significant corrosion. Moreover, the fact that only H_2_, but no PH_3_ was detected by OLEMS (Figure S3b) during an applied potential sweep means that CoP_*x*_ dissolution most likely does not proceed via a Co^δ+^ reduction route as observed for the electrochemical dissolution of other metal phosphides (e. g. InP+3H^+^+3e^−^→In^0^+PH_3_↑).^39^ This observation is in agreement with the hypothesis of Costa *et al*. who proposed for NiP_2_ that an anodic dissolution mechanism should be more likely than a cathodic degradation (NiP_2_+2H^+^+3O_2_+2 H_2_O→Ni^2+^+2H_3_PO_4_).^35^ A similar mechanism may thus be expected for the present CoP_*x*_ case. Adding to the preceding compositional analysis, an SEM and AFM analysis of the CA‐treated electrode reveals moderate pitting of the surface (Figure S8c, d). On the other hand, the rLSV‐treated sample was subjected to a repeated sequence of potential sweeps in which the potential was gradually changed from OCP (*E*=+0.24 V) to HER potential (*E*=−0.26 V) with a sweep rate of 20 mV/s (25 s per scan). Dissolution during the rLSV treatment can be partially explained by minor anodic currents occurring at higher potentials (Figure S10). However the major part of Co (105 nmol/cm^2^) was dissolved in cathodic region of LSV and with that is still higher than that of CA treatment (13 nmol/cm^2^). This might be explained by the fact that such a frequent change of load (i. e. *j*_start_≈0 mA/cm^2^, *j*_end_≈−5 mA/cm^2^) creates additional stress to the electrode by forming reverse currents upon repeated sweeping. A similar phenomenon is widely observed in commercial electrolyzers and fuel cells which suffer from reverse (or shunt) currents created by the shut‐down/start‐up of electrolyzer.^61^,^62^ Reverse currents might originate from the oxidation of H_2_ produced during the preceding HER and accompanied by anodic degradation of the electrode. Indeed, in case of a constant cathodic treatment, no additional electrode corrosion was observed since the electrode was subjected to a constant negative current.

Another important parameter which characterizes HER performance of CoP_*x*_ is its faradaic efficiency. We conducted OLEMS measurements in order to evaluate the change in CoP_*x*_ faradaic efficiency upon constant and interrupted electrolysis, similar to the performed CA and rLSV experiments. Faraday plots represent Faraday's law of electrolysis and can be used to detect possible electrode (de)activation processes related to surface changes. In the ideal case of a stable electrode, Faraday plots of the same electrode surface must have the same Faraday slope independent of the applied treatment (i. e. faradaic and gas collection efficiencies are constant). However, in cases where the surface of the electrode overcomes transformations or other side‐reactions begin to contribute to the measured faradaic charge, faradaic efficiency of the main reaction will also change resulting in a deviation of the Faraday slope.^38^ We conducted two experiments under conditions similar to CA and rLSV, but slightly modified to fit the requirements of OLEMS.

**Figure 8:**
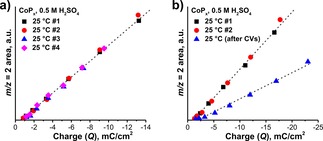
Faradaic efficiency of CoP_*x*_ in 0.5 M H_2_SO_4_ (charge vs. H_2_^+^ ion current) under a) consecutive HER and b) HER with intermittent CVs.

In the first experiment, conforming CA, a fresh CoP_*x*_ was subjected to a series of consecutive potential pulses (*E*=0.0→−0.16 V, in 20 mV steps) repeated 4 times. Short repeated pulses were preferred over continuously applied potential since OLEMS cannot assure an adequate linear detection of large quantities of evolved H_2_. The plot shows a reproducible dependence between charge and evolved H_2_, which can be interpreted as constant faradaic efficiency for HER (Figure 8a). Despite electrode dissolution, we can conclude that upon CA treatment CoP_*x*_ preserves its structure and also the number of involved HER‐active sites is unaffected. In this case, FE should be preserved until the critical point at which a less HER‐active surface (e. g. substrate) starts to be exposed to the electrolyte as well.

In the second experiment, fresh CoP_*x*_ was first subjected to a series of consecutive potential pulses (*E*=0.0→−0.16 V, in 20 mV steps) repeated twice to validate the previously observed constant FE. Then, a series of cyclic voltammograms (CVs, +0.24→−0.12 V, 20 mV/s, 65x) was applied to initiate changes similar to what was observed in the rLSV experiment and subsequently, another Faraday plot was recorded (Figure 8b). For the first two consecutive scans (black and red symbols) the Faraday slopes were found to be the same, as it was expected from the previous experiment. However, after the electrode was subjected to a series of CVs, a significant decrease of the FE was observed (blue symbols). This phenomenon can be explained by surface transformation (assumingly oxidation) of the CoP_*x*_ during the potential sweep, which results in a lower FE due to the lower HER activity of formed CoO_*x*_ compounds. This hypothesis goes along with the previously observed enhanced dissolution of the rLSV‐treated electrode.

## 3 Conclusions

In this work, we synthesized model CoP_*x*_ electrodes by thermal phosphidation of flat PE‐ALD‐fabricated Co_3_O_4_ thin films. Chemical and structural analysis of the films revealed two major fractions composing the sample: CoP in the bulk and a P‐rich (P/Co>1) CoP_*x*_ phase at the surface. Formation of the P‐rich phase in the top‐most 23 % of the film was observed by XPS depth profiling and was attributed to a diffusion limitation of P during the thermal phosphidation resulting in a P gradient. Formation of a bulk CoP phase was also confirmed by Raman and validated by additional DFT calculations and XRD experiments. Morphology analysis confirmed roughening and agglomeration of the initial Co_3_O_4_ film upon thermal phosphidation. The exchange current density of the electrode was found to be comparable with reported values (*j*_0_=8.9 ⋅ 10^−5^ A/cm^2^) and the apparent activation energy for HER (*E*_app_=81±15 kJ/mol) of this system was measured for the first time. The large value of the Tafel slope (∼100 mV/dec) together with the fact that P is the HER‐active H^+^‐adsorbing site suggested that the reaction is limited by the number of P sites.

A detailed stability study revealed that the material is unstable in 0.5 M H_2_SO_4_ with a constant dissolution rate (Co) of 2.4 nmol h^−1^ cm^−2^ even when no additional electrochemical treatment was applied. The constant cathodic treatment (CA) was found to have no influence on the dissolution rate, meaning that dissolution does not involve cathodic corrosion of CoP_*x*_. Moreover, OLEMS could not confirm the formation of PH_3_ upon cathodic potential cycling. The faradaic efficiency towards HER was found to be constant within ±10 % when a protective cathodic current was applied in between the temperature steps. However, an interruption under resting conditions (OCP) resulted in a significant decrease of the faradaic efficiency. These findings confirm that an anodic corrosion of CoP_*x*_ is prevailing in accordance with literature. Dynamic potential changes performed in a repeated way (repeated cathodic linear sweep voltammetry) were found to increase dramatically the dissolution rate of the film leading to its complete degradation in 5 hours. This observation was explained by the fact that frequent change between HER and open circuit potentials increases the electrochemical stress applied to the electrode yielding a flow of transient reverse (i. e. anodic) currents which facilitate oxidation of CoP_*x*_. A similar issue is commonly detected in commercial electrolyzers upon electrolysis interruption, but was not yet observed for TMP systems.

In this study, we show the importance of employing a multi‐technique characterization approach for an unambiguous evaluation of electrode stability. We stress out and showcase that current density constancy is not necessarily indicative for electrode stability and changes of faradaic efficiency cannot be accurately reflected in chronoamperometric measurements solely. Moreover, we show that the assessment of the chemical stability of electrodes under standby conditions is as important as stability under applied potentials.

## **Experimental Section**

### Co_3_O_4_ PE‐ALD

20 nm thick Co_3_O_4_ films were grown on commercial Au(100 nm)/Ti/Si(100) (Sigma Aldrich) substrates by plasma‐enhanced atomic layer deposition (PE‐ALD). CoCp_2_ (98 %, Strem Chemicals) was used as Co precursor and an O_2_ plasma (100 W, 0.01 bar) was used as oxidant. The deposition was carried out at 100 °C. The growth rate of Co_3_O_4_ was set to 0.05 nm/cycle. A single deposition cycle was composed of the following steps: precursor dosing (4 s), purging (6 s), plasma treatment (8 s), and second purging (6 s). No additional post‐treatment was applied to as‐deposited Co_3_O_4_ films. More details on the technical parameters of the utilized PE‐ALD setup and Co_3_O_4_ deposition process are given elsewhere.^63^

### Phosphidation Procedure

Phosphidation of PE‐ALD fabricated Co_3_O_4_ films was carried out in a horizontal calcination tube furnace. The substrates with an average area of 1 cm^2^ were positioned in the middle of the tube together with a quartz boat containing 0.5 g of sodium hypophosphite monohydrate NaH_2_PO_2_⋅H_2_O (Sigma Aldrich, 99 %) used as P‐precursor. The boat was located upstream relative to the gas flow consisting of 4 : 1 Ar/H_2_, 150 mL/min STP. The reactor was heated to 260 °C (20 °C/min) to dehydrate the sodium hypophosphite followed by a further temperature increase to the target *T* (350–450 °C) with a slower heating rate (2 °C/min). The reactor was then kept at the target *T* for 30 min. Upon thermal decomposition, sodium hypophosphite releases PH_3_ which further reacts with Co_3_O_4_ to form cobalt phosphide. Unreacted phosphine was collected at the outlet by a liquid trap containing a concentrated aqueous CuSO_4_ solution. The sample was let to cool to room temperature in the same gas composition. Thermally treated substrates were rapidly transferred through air and stored in a glovebox. Such transfer results in the formation of a thin native passivation layer composed of PO_4_^3−^ and P^0^ species.

### Electrochemical Cell and On‐line Electrochemical Mass Spectrometry (OLEMS)

In our experiments, we used a standard water jacketed electrochemical (EC) glass cell equipped with an OLEMS tip. CoP_*x*_/Au/Ti/Si substrates with an average size of 0.1 cm^2^ were fixed on FTO glass slides (1.5×0.5 cm^2^) by conductive Ag paint (RS components) with additional top‐contact. To avoid the electric response of substrate and Ag, all conductive surfaces except CoP_*x*_ were covered with a layer of insulating epoxy (Henkel, LOCTITE EA9492). The working electrode (WE) slides were mounted into a PEEK holder and contacted from the top by a gold wire enclosed in a quartz tube. The holder was additionally covered by several layers of Teflon tape (Swagelok) to prevent electrolyte interaction with the contact wire. The OLEMS tip was made of a PEEK tube with a porous hydrophobic Teflon plug inside and fixed in another quartz tube. The OLEMS tip was approached 20 μm above the surface of the working electrode by the micrometer screw with help of a video microscope (Figure S11a). A Pt plate (10 cm^2^) and a Red Rod electrode (Radiometer Analytical, *E*_RE_=+0.220 V) were utilized as counter (CE) and reference (RE) electrodes, respectively, and connected to an Ivium Compactstat potentiostat (Ivium Technologies). Ar‐purged 0.5 M H_2_SO_4_ (Sigma Aldrich, 99.999 %) was used as electrolyte. Before the experiments, the EC cell was cleaned with chromic acid (85 mmol K_2_Cr_2_O_7_+9.2 mol H_2_SO_4_). Milli‐Q water (18.2 MΩ ⋅ cm) was used in all cleaning and dilution steps. The temperature of the cell (25–35 °C) was maintained by a thermostat.

All potentials reported in this work are plotted on *iR*‐corrected reversible hydrogen electrode (RHE) scale, if not mentioned otherwise and current densities are normalized on the geometric surface area of the electrodes. More detailed information on the cell construction and OLEMS operation principles are given elsewhere.^38^,^40^

### Stability of CoP_x_ under Acidic HER Conditions

HER conditions in acidic electrolyte, a series of experiments was performed. In the first experiment, a fresh CoP_*x*_ electrode was continuously polarized at *E*=−0.12 V for 5 h and the electric current was recorded (chronoamperometry, CA). In the second experiment, a series of linear sweep voltammograms was repeatedly applied to a fresh CoP_*x*_ electrode (repeated LSV=rLSV). The potential range of voltammograms was selected starting from +0.24 V (open circuit potential, OCP) till −0.26 V with a scan rate of 20 mV/s. The total duration of the rLSV experiment was also set to 5 h. In the third (reference) experiment, a fresh CoP_*x*_ substrate was immersed into 0.5 M H_2_SO_4_ for 5 h without additional EC treatment. The (electro)chemical stability of the electrodes was then evaluated by ICP‐OES, XPS and Raman spectroscopy.

### Measurements of HER Kinetics on CoP_x_

*T*‐dependent kinetic measurements were performed on a fresh CoP_*x*_ electrode. The electrode was subjected to a series of cathodic potential pulses ranging from the resting potential (−0.20 V vs. Red Rod) till *E*=−0.40 V vs. Red Rod in 20 mV steps (Figure S11b). Each pulse was applied for 10 s and the time between separate pulses was kept at 60 s to remove excess of produced H_2_. During a temperature change, a protective potential (−0.36 V vs. Red Rod) was applied for 40 min. The same potential sequence was repeated for each temperature (25 °C, 28 °C, 31 °C, 35 °C). The temperature of the electrolyte was measured by a thermometer positioned near the WE. The temperature range was limited to 35 °C to reduce possible electrode degradation by temperature increase. Potentials were later normalized on the thermal drift of the RE and are reported on the RHE scale. Along with electrochemical treatment, OLEMS ion currents of the following *m/z* values were continuously recorded: 2 (H_2_^+^), 28 (N_2_^+^), 31 (P^+^), 32 (O_2_^+^ and PH^+^), 33 (PH_2_^+^) and 34 (PH_3_^+^). For comparison, the same experiment was repeated without applying a protective potential.

In order to evaluate the change of faradaic efficiency (FE) under facilitated dissolution conditions (rLSV), a series of cyclic voltammograms was applied prior to the kinetic measurements. Constant FE of yet untreated sample was first validated by recording two consecutive Faraday curves which showed the same values of the slope. Voltammetric treatment led to a decrease of Faraday slope.

### Sample Characterization

Inductively Coupled Plasma Optical Emission Spectrometry (ICP‐OES) measurements were performed on a SPECTROBLUE EOP spectrometer equipped with an axial plasma source (Ar). Sample uptake rate was set to 2 ml/min. The emission intensity of Co ions was measured at 228.6 and 238.9 nm. The content of P was found to be below the detection limit of the spectrometer.

X‐ray photoelectron spectroscopy (XPS) measurements were carried out on a K‐Alpha XP spectrometer (Thermo Scientific), equipped with a monochromatic small‐spot (400 μm) X‐ray source, a 180° double focusing hemispherical analyzer with a 128‐channel detector and an Al anode (Al *K*α=1486.6 eV). The background pressure inside the analysis chamber was kept below 8 ⋅ 10^−8^ mbar reaching maximum of 3 ⋅ 10^−7^ mbar during the measurements due to the flow of low energy Ar^+^ ions involved in the charge neutralization process. High‐resolution spectra of core levels (Co 2p, P 2p, Au 4f, Au 4d_5/2_) and wide‐range survey spectra were recorded with a pass energy of 50 eV and 200 eV, respectively. Due to significant overlap between Au 4f peaks and the Co 3p loss feature, the Au 4d_5/2_ level was used for quantification instead. XP spectra were taken at the different positions of the surface to average the obtained quantitative results. XPS depth profiles were recorded on the same spectrometer. Ar^+^ sputtering (*p*=1 ⋅ 10^−7^ mbar) was done at 1 kV with time steps of 10 s (for the surface region) and 30 s (for bulk). XP spectra were taken after each sputtering step. XP spectra of spent electrodes were taken shortly after performed treatments. Electrodes were dismounted from the EC cell, rinsed with copious amounts of Milli‐Q water, dried with compressed air and immediately introduced to the vacuum chamber of an XP spectrometer. Total duration of air exposure was estimated to be approximately 15 min (before and after each experiment) (Figure S12).

Raman spectra were recorded on a Horiba Raman microscope equipped with an Olympus LMPlanFl 100x 0.80 lens and HeNe laser (632.8 nm) with a diffraction grating of 600 gr/mm. Each spectrum was collected for 20 s (2×10 s).

Tapping mode atomic force microscopy (AFM) measurements were carried out on NT‐MDT Next microscope. Both height and phase images were recorded simultaneously with gold‐coated Si probes with a curvature radius of 10 nm (NSG10, NT‐MDT). Scanning electron micrographs (SEM) were recorded on a FEI Quanta 3D FEG microscope at an accelerating voltage of 5 kV without additional coating of the surface.

## Conflict of interest

The authors declare no conflict of interest.

## Supporting information

As a service to our authors and readers, this journal provides supporting information supplied by the authors. Such materials are peer reviewed and may be re‐organized for online delivery, but are not copy‐edited or typeset. Technical support issues arising from supporting information (other than missing files) should be addressed to the authors.

Supplementary
