# Supplementary material for: Stability of CoPx Electrocatalysts in Continuous and Interrupted Acidic Electrolysis of Water
Source: ChemElectroChem. 2018 Feb 22;5(8):1230–9. doi: 10.1002/celc.201701119 (PMC5915747; doi:10.1002/celc.201701119)
Supplement: Supplementary file 1 — Supplementary [file CELC-5-1230-s001.pdf]

Characterization of Co_3O_4 and CoP_x films:

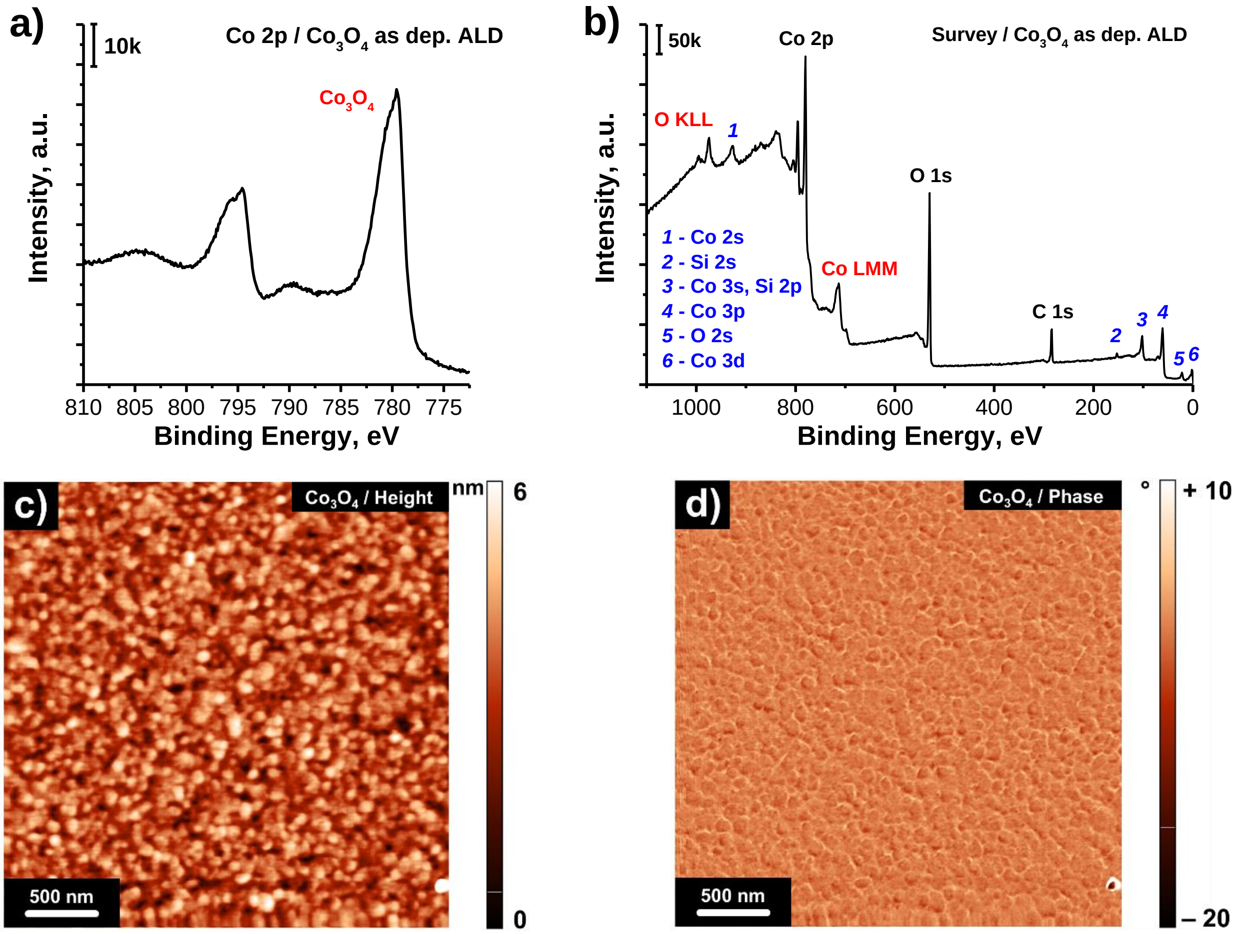

Figure S1. a) Co 2p and b) survey XP spectra of as deposited (PE-ALD) Co_3O_4; c) height and d) phase atomic force micrographs of as-prepared Co_3O_4 film recorded in tapping mode.

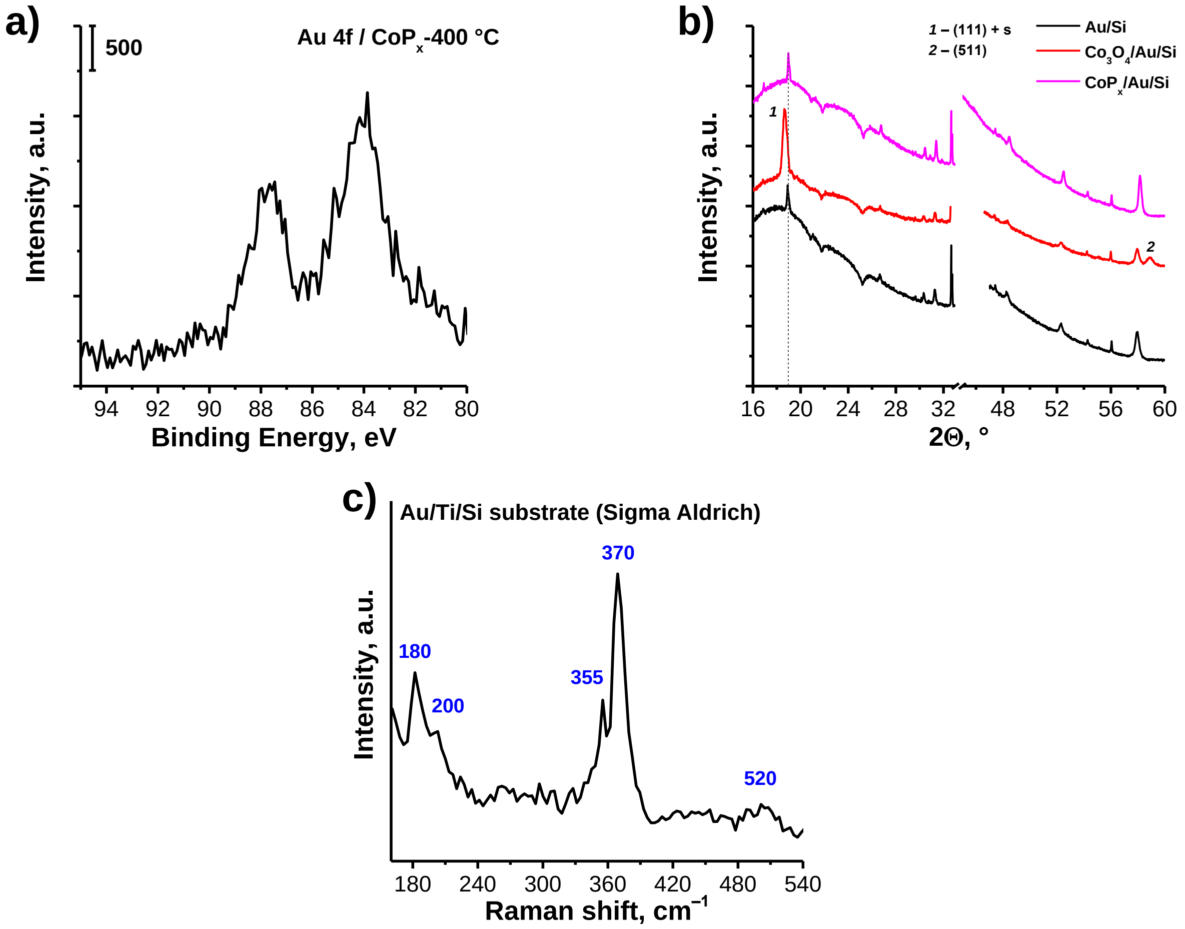

Figure S2. a) Au 4f XP spectrum of a fresh CoP_x (400 °C) film (substrate); b) XRD patterns of the substrate, Co_3O_4 and CoP_x films; c) Raman spectrum of the bare cleaned Au/Ti/Si substrate.

Peaks at 180, 200, 355 and 370 cm^{-1} correspond to HeNe laser impurities such as: contribution of additional laser modes and Raman lasing (Figure S2 c).[1,2] The peak at 520 cm^{-1} can be attributed to Si of the Au/Ti/Si substrate.

Performance and EC stability of CoP_x electrodes:

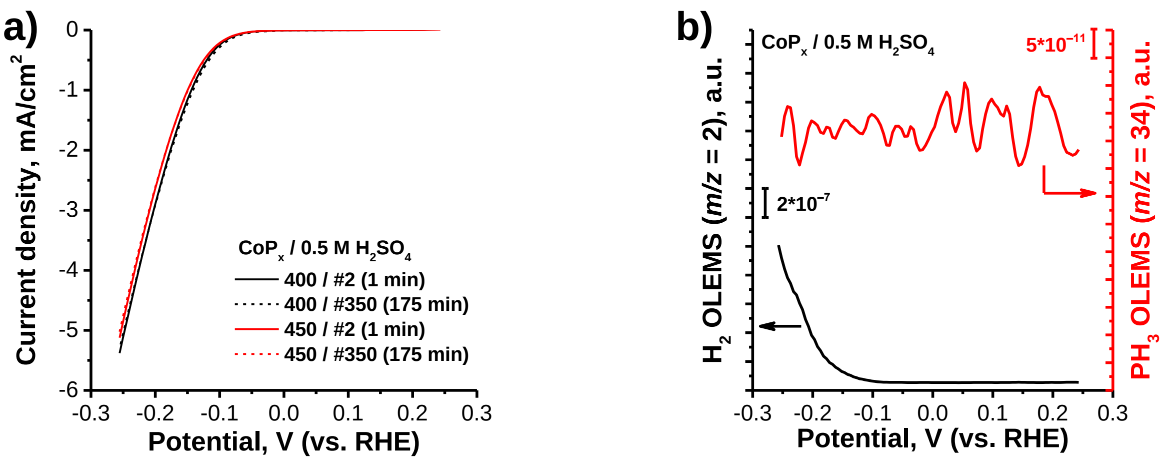

Figure S3. a) Comparison of activity and stability between CoP_x cathodes prepared at different temperatures; b) potential-dependent $m/z = 2$ (H_2^+) and $m/z = 34$ (PH_3^+) ion currents recorded on CoP_x during cathodic potential scan in 0.5 M H_2SO_4.

Bulk and surface chemical composition of CoP_x:

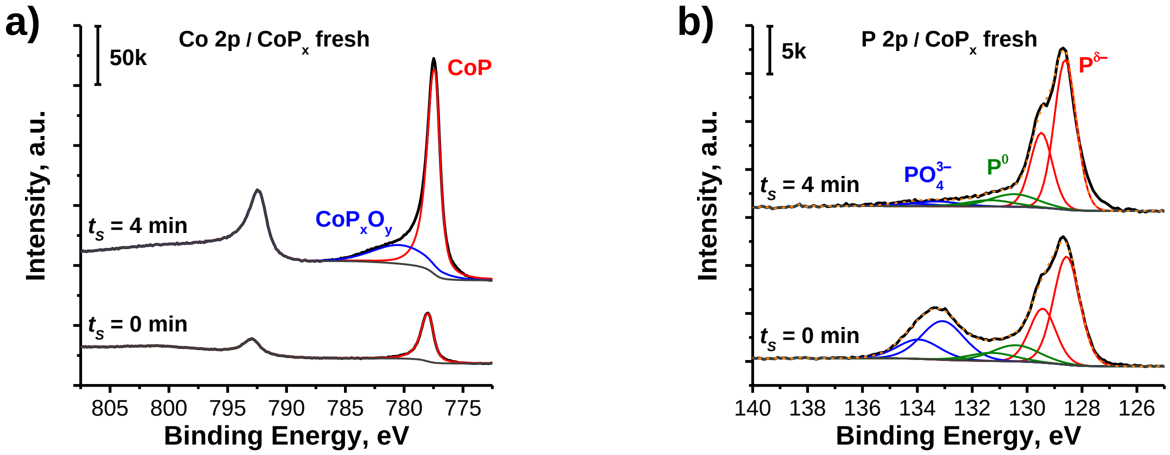

Figure S4. a) Co 2p and b) P 2p XP spectra of fresh CoP_x electrodes before and after 4 min of sputtering (see Figure 3 a).

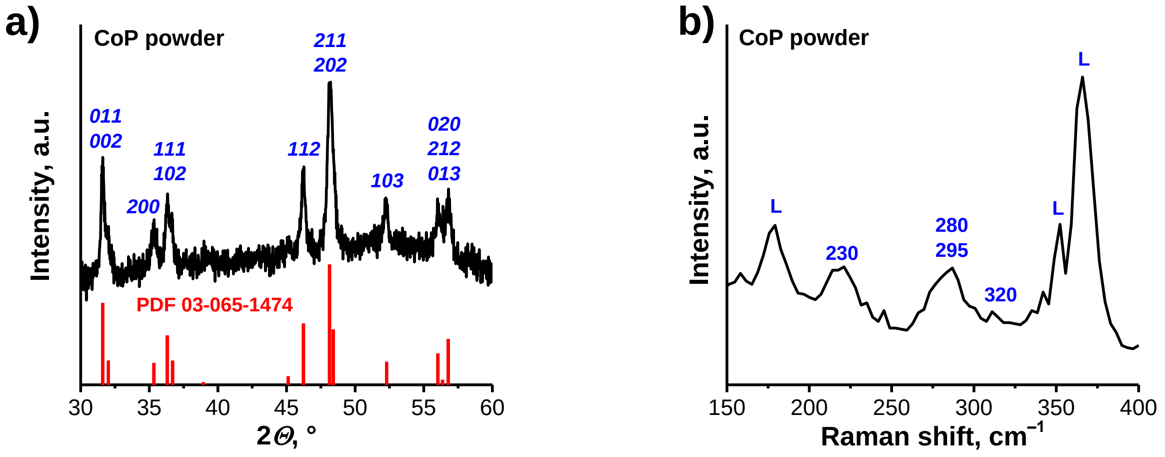

Figure S5. a) XRD pattern and b) Raman spectrum (λ_{ex} = 632.8 nm) of bulk CoP prepared by thermal phosphidation of Co_3O_4 powder, where L marks spectral impurities of the HeNe laser.

DFT calculation of CoP structure and Raman spectrum:

To calculate the Raman spectrum of the CoP crystal structure (orthorhombic, Pnma (62)), a two-step procedure was followed. At first, spin-polarized optimization of the CoP crystal structure was performed using periodic density functional theory (DFT) with the PBE exchange-correlation functional[3] as implemented in the Vienna Ab-Initio Simulation Package (VASP).[4–8] The electronic wavefunctions were expanded following the projected-augmented-wave scheme (PAW) to describe the electron-ion interactions. Integration in the first Brillouin zone was performed using a 7x7x7 Monkhorst-Pack k-point mesh. The total energies were computed with a cut-off energy of 400 eV and a root-mean-square (RMS) force convergence criterion of 0.01 eV/Å. Next, the unit cell was expanded in the b-direction such that a 1x2x1 supercell with the chemical composition Co_8P_8 was obtained. The atom positions were used directly in Gaussian 09 Rev. A0.2 [9] to compute vibrational frequencies and intensities of the Co_8P_8 cluster without prior geometry optimization. The optimization step was omitted in order to avoid deformation of the Co_8P_8 cluster due to it not being part of a periodic structure anymore. The vibrational frequencies and intensities were calculated using the PBE exchange-correlation functional and the 6-311g all-electron basis set. The exact procedure to obtain the Raman spectrum is outlined elsewhere.[10] Calculated intensities should be considered qualitative.

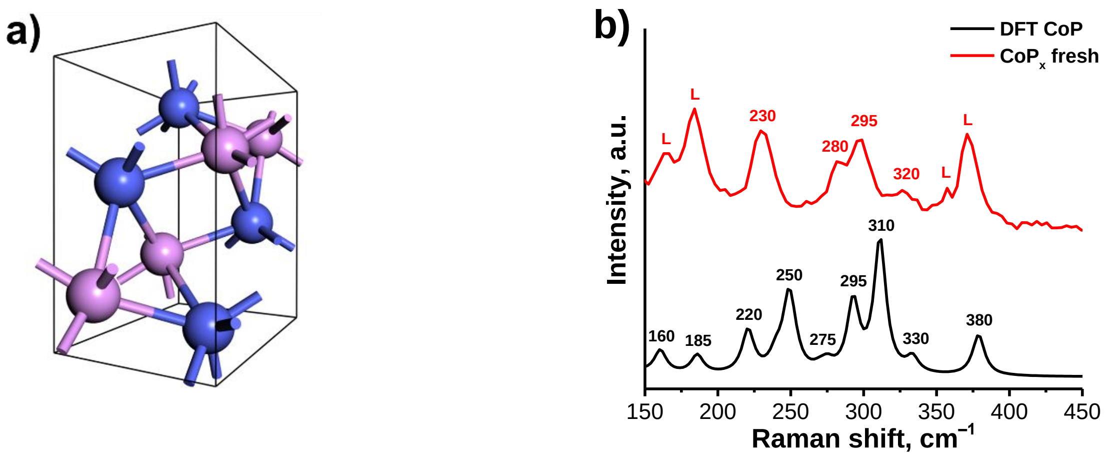

Figure S6. a) Co_4P_4 unit cell and b) simulated (black) Raman spectrum of a Co_8P_8 cluster in comparison with experimental Raman spectrum of CoP_x film (red).

Effect of protective current on HER Faradaic efficiency of CoP_x:

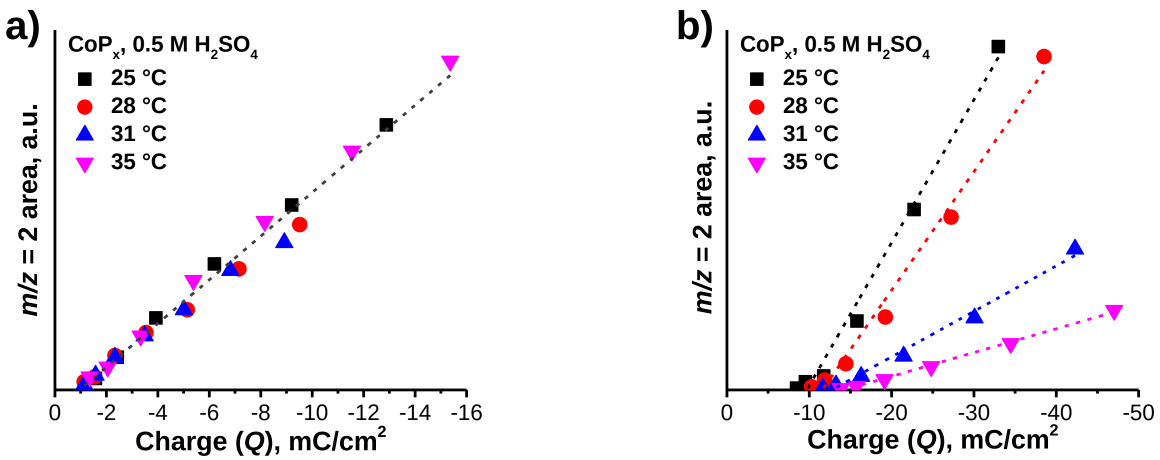

Figure S7. a) Faraday plots of HER performed on CoP_x in 0.5 M H_2SO_4 a) with and b) without protective CA in-between.

Characterization of spent CoP_x electrodes:

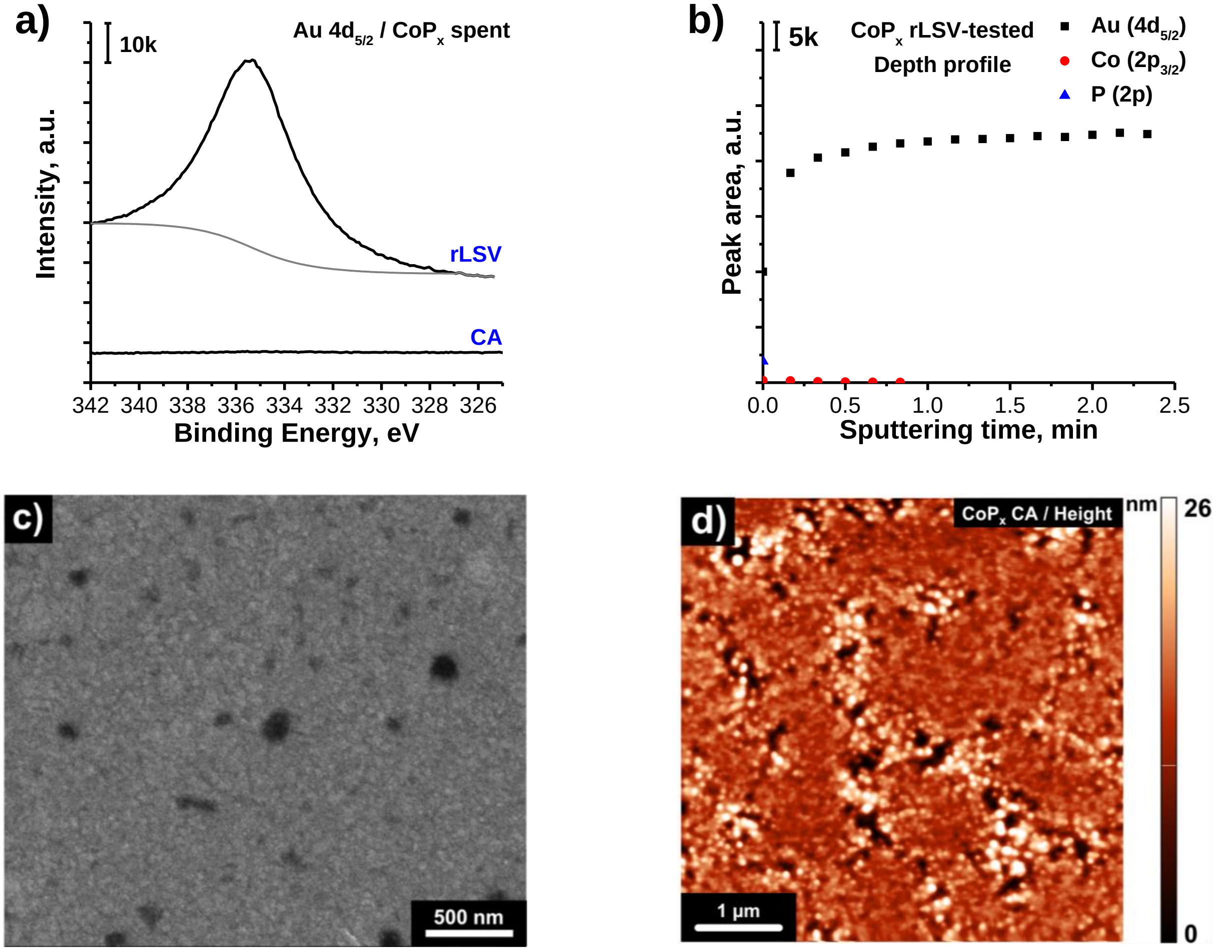

Figure S8. a) Au $4d_{5/2}$ XP spectra of CA and rLSV treated CoP_x electrodes; b) DP-XPS of rLSV-treated CoP_x; c) SE and d) AF micrographs of CA-treated CoP_x.

Co dissolution during CA treatment:

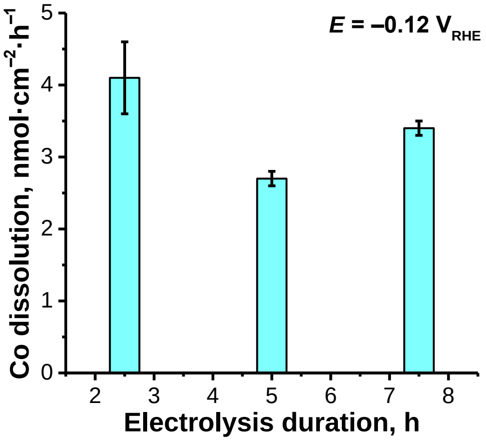

Figure S9. ICP-OES evaluation of Co dissolution rate during CA treatment of CoP_x electrode in 0.5 M H_2SO_4.

A fresh CoP_x electrode was subjected to a constant CA treatment at E = –0.12 V. In order to evaluate the temporal evolution of Co dissolution, the electrolyte was sampled after 2.5 h, 5.0 h and 7.5 h of electrolysis. Dissolution rates were evaluated by ICP-OES. Initial dissolution rates (CA < 2.5 h) could not be determined by the available instrumentation due to the low concentration of Co ions. However, a more accurate evaluation of initial dissolution rates could be done by utilizing for instance online ICP methods such as online ICP-MS connected to a flow cell.[11]

Estimation of anodic CoP_x dissolution during rLSV treatment:

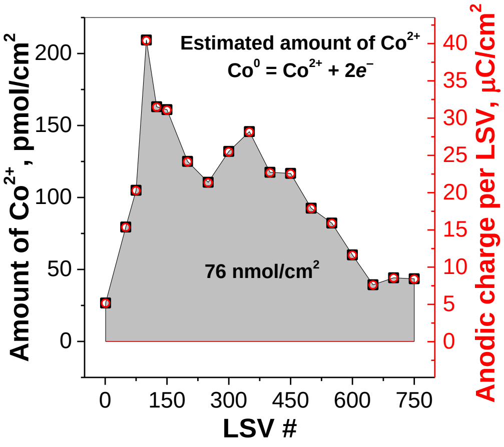

Figure S10. Anodic charge and estimated amount of Co^{2+} produced per LSV in the rLSV treatment of CoP_x conducted in 0.5 M H_2SO_4.

The amount of dissolved Co (n_{Co}) was estimated from the values of anodic charge (Q_{Co}) generated in the rLSV treatment. Values of anodic charge were derived from the separate LSVs of the rLSV experiment by integration of the anodic currents. Furthermore, anodic charge was converted to estimated quantities of Co^{2+} via Faraday's law of electrolysis ($n_{Co} = 0.5 \cdot Q_{Co} \cdot F^{-1}$) by assuming the following faradaic reaction: $Co^0 \rightarrow Co^{2+} + 2e^-$.

OLEMS scheme and kinetic measurements procedure:

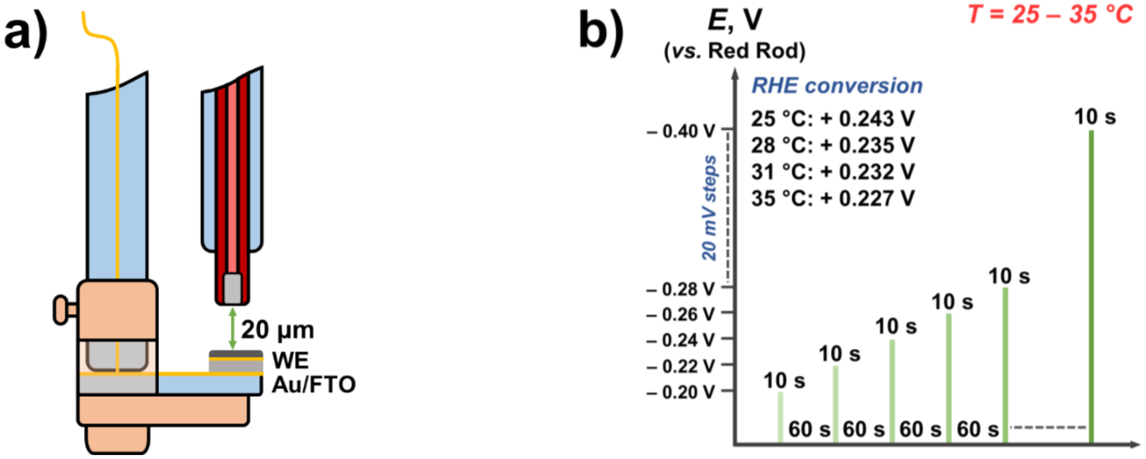

Figure S11. a) Scheme of the WE-tip geometry used in the OLEMS measurements; b) scheme of temperature-dependent kinetic experiments.

Influence of exposure to air on CoP_x composition:

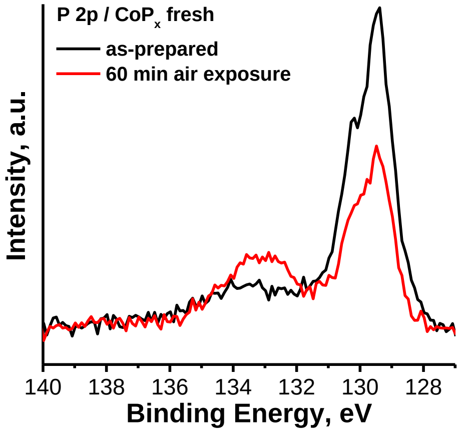

Figure S12. P 2p XP spectra of a fresh CoP_x electrode before and after 60 min of exposure to ambient air.

CoP_x was subjected to ambient air oxidation for 60 min. The duration of air exposure was deliberately chosen to be significantly longer (60 min) than in the actual experiments (~15 min). The increase of $InPO_x$ content (BE = 138-131 eV) is due to surface oxidation upon air exposure.

References

[1] M. Endo, R. F. Walter, *Gas Lasers*, CRC Press, **2006**.

[2] J. Assendrup, B. Grover, L. Hall, S. Jabr, *Appl. Phys. Lett*. **1986**, *48*, 86–88.

[3] B. K. Perdew, J. P. M. Ernzerhof, *Phys. Rev. Lett*. **1996**, *77*, 3865–3868.

[4] G. Kresse, J. Hafner, *Phys. Rev. B* **1994**, *49*, 14251–14269.

[5] G. Kresse, J. Furthmüller, *Phys. Rev. B* **1996**, *54*, 11169–11186.

[6] G. Kresse, J. Furthmüller, *Comput. Mater. Sci*. **1996**, *6*, 15–50.

[7] G. Kresse, D. Joubert, *Phys. Rev. B* **1999**, *59*, 1758–1775.

[8] G. Kresse, J. Hafner, *Phys. Rev. B* **1993**, *47*, 558–561.

[9] Gaussian 09, Revision A.02, M. J. Frisch, G. W. Trucks, H. B. Schlegel, G. E. Scuseria, M. A. Robb, J. R. Cheeseman, G. Scalmani, V. Barone, G. A. Petersson, H. Nakatsuji, X. Li, M. Caricato, A. Marenich, J. Bloino, B. G. Janesko, R. Gomperts, B. Mennucci, H. P. Hratchian, J. V. Ortiz, A. F. Izmaylov, J. L. Sonnenberg, D. Williams-Young, F. Ding, F. Lipparini, F. Egidi, J. Goings, B. Peng, A. Petrone, T. Henderson, D. Ranasinghe, V. G. Zakrzewski, J. Gao, N. Rega, G. Zheng, W. Liang, M. Hada, M. Ehara, K. Toyota, R. Fukuda, J. Hasegawa, M. Ishida, T. Nakajima, Y. Honda, O. Kitao, H. Nakai, T. Vreven, K. Throssell, J. A. Montgomery, Jr., J. E. Peralta, F. Ogliaro, M. Bearpark, J. J. Heyd, E. Brothers, K. N. Kudin, V. N. Staroverov, T. Keith, R. Kobayashi, J. Normand, K. Raghavachari, A. Rendell, J. C. Burant, S. S. Iyengar, J. Tomasi, M. Cossi, J. M. Millam, M. Klene, C. Adamo, R. Cammi, J. W. Ochterski, R. L. Martin, K. Morokuma, O. Farkas, J. B. Foresman, and D. J. Fox, Gaussian, Inc., Wallingford CT, **2016**.

[10] D.-Y. Wu, X.-M. Liu, S. Duan, X. Xu, B. Ren, S.-H. Lin, Z.-Q. Tian, *J. Phys. Chem. C* **2008**, *112*, 4195–4204.

[11] M. Ledendecker, J. S. Mondschein, O. Kasian, S. Geiger, D. Göhl, M. Schalenbach, A. Zeradjanin, S. Cherevko, R. E. Schaak, K. Mayrhofer, *Angew. Chemie Int. Ed.* **2017**, *56*, 9767–9771.
